# Patients With Rheumatoid Arthritis Increased Risk of Developing Osteoarthritis: A Nationwide Population-Based Cohort Study in Taiwan

**DOI:** 10.3389/fmed.2020.00392

**Published:** 2020-09-10

**Authors:** Yung-Heng Lee, Hsi-Kai Tsou, Su-Ling Kao, Shuo-Yan Gau, Yi-Chiao Bai, Mei-Chen Lin, James Cheng-Chung Wei

**Affiliations:** ^1^Department of Health Services Administration, China Medical University, Taichung, Taiwan; ^2^Department of Public Health, China Medical University, Taichung, Taiwan; ^3^Department of Orthopedics, Cishan Hospital, Ministry of Health and Welfare, Kaohsiung, Taiwan; ^4^Department of Center for General Education, National United University, Miaoli, Taiwan; ^5^Functional Neurosurgery Division, Neurological Institute, Taichung Veterans General Hospital, Taichung, Taiwan; ^6^Department of Rehabilitation, Jen-Teh Junior College of Medicine, Nursing and Management, Houlong, Taiwan; ^7^Department of Human Resource, Cishan General Hospital, Kaohsiung, Taiwan; ^8^Institute of Medicine, Chung Shan Medical University, Taichung, Taiwan; ^9^Management Office for Health Data, China Medical University Hospital, Taichung, Taiwan; ^10^College of Medicine, China Medical University, Taichung, Taiwan; ^11^Department of Rheumatology, BenQ Medical Center, The Affiliated BenQ Hospital of Nanjing Medical University, Taichung, Taiwan; ^12^Institute of Medicine, Chung Shan Medical University, Taichung, Taiwan; ^13^Department of Allergy, Immunology and Rheumatology, Chung Shan Medical University Hospital, Taichung, Taiwan; ^14^Graduate Institute of Integrated Medicine, China Medical University, Taichung, Taiwan

**Keywords:** rheumatoid arthritis, osteoarthritis, population-based cohort study, autoimmue disease, chronic inflammation

## Abstract

**Objective:** To investigate the risk of developing OA in patients diagnosed with RA.

**Methods:** In this study, we presented gender, age, urbanization, occupation and, comorbidities in a RA cohort and a non-RA cohort based on number and percentage. We investigated the OA risk in patients with RA. We conducted a retrospective cohort study with a 13-year longitudinal follow-up in Taiwan. Patients who received RA diagnoses between 2000 and 2012 were enrolled in the study cohort. The non-RA cohort were 1:1 propensity score matched with the RA cohort by age, gender, index year, urbanization, occupation, and comorbidities. The hazard ratios (HRs) and adjusted HRs (aHRs) were estimated after confounders were adjusted. Sensitivity analysis utilizing the Longitudinal Health Insurance Database (LHID) was conducted.

**Results:** We totally enrolled 63,626 cases in RA patients (study cohort) and matched controls. In the RA cohort, the crude HR for OA was 2.86 (95% confidence interval (CI), 2.63–3.11, *p* < 0.001), and the aHR was 2.75 (95% CI, 2.52–2.99, *p* < 0.001). (The study demonstrated that patients with RA had a higher risk for developing OA compared with the non-RA controls.

**Conclusion:** Developing effective OA prevention strategies are necessary in patients with RA. This finding may be extended to evaluate the risk of OA among other kinds of inflammatory autoimmune diseases. Identifying the key pathogenesis mechanisms are necessary in the future study.

## Introduction

Rheumatoid arthritis (RA) is an autoimmune disease characterized by chronic inflammation that can cause joint destruction. It may interfere with daily activities and have a serious adverse effect on quality of life (1). RA patients usually show painful symmetrical joint swelling and warmth, and morning stiffness (2). In Asia, the average age of onset of RA is 50-years-old, affecting 0.1–0.3% of the general population, predominately women (3). The average age-adjusted annual incidence rate is 15.8 per 100,000 people in Taiwan and the adjusted incidence rate is 20.9–25.2 and 7.0–8.2 per 100,000 person-years in women and men, respectively (4). The pathogenesis of RA is very complex, involving environmental factors and genetic factors (5). About half of RA patients experience specific serological abnormalities several years before symptoms appear (6). RA is characterized by highly vascularized synovitis, which leads to bone erosion, cartilage damage, and joint destruction (7). Inflammatory cytokines, such as tumor necrosis factor alpha (TNFα), interleukin (IL)-1, and IL-6, play a crucial role in the pathogenesis of RA (8).

Osteoarthritis (OA) is a common musculoskeletal disease (9). OA affects 240 million people worldwide, ~10% of men and 18% of women over 60 years of age suffer from OA (10). OA is the most common joint disorder in the United States (11). For Taiwanese between the ages of 65 and 84, the self-reported male prevalence rate is 20–24%, and the female is 34–35% (12). OA is originally thought to be a degenerative joint disease caused by “wear,” but the current perception of the disease is that inflammation may also be one of the key factors in the occurrence and progression of OA (13). Some inflammatory cytokines are involved in the pathophysiological events in OA (14). The most important mediators controlling OA appear to be inflammatory cytokines, including IL-1β, TNFα, IL-6, IL-15, IL-17, and IL-18 as well as anti-inflammatory cytokines such as IL-4, IL-10, and IL-13 (13).

According to the above literature review, chronic inflammation is one of the major causes in both RA and OA. Chronic inflammation can also cause damage to cartilage and soft tissue, followed by joint instability. It seems to create a link between RA and OA disorders. We hypothesized that patients with RA may have an increased risk of developing OA. This longitudinal retrospective population-based cohort study aims to investigate the risk of developing OA in patients diagnosed with RA using the National Health Claims Database in Taiwan.

## Experimental Section

### Data Sources and Ethics Approval

In 1995, the Taiwanese government established a database called the National Health Insurance Research Database (NHIRD), which contains a history of outpatients, inpatients, medical treatments, and medication prescriptions. As of today, more than 99% of Taiwanese citizens are registered in the database. The database is encrypted for privacy before it is released for research.

In this study, we conducted analysis of the population-based inpatients file from 1996 to 2013 based on NHIRD. The diagnoses are coded according to the International Classification of Disease, Ninth Revision, Clinical Modification (ICD-9-CM). In sensitivity analysis, a subset of NHIRD, the Longitudinal Health Insurance Database (LHID) is applied. The study was approved by the Research Ethics Committee of China Medical University and Hospital in Taiwan (CMUH). The approval number is CMUH-104-REC2-115-R3.

### Study Subjects

To clarify the association between RA and OA, we defined two cohorts in this study: the RA cohort and non-RA cohort. Patients who were newly diagnosed and hospitalized by RA (ICD-9-CM code: 714.0) and had received catastrophic illness cards between 2000 and 2012 were identified as RA patients. The application date for a catastrophic illness card was set as the index date. The non-RA cohort were 1:1 propensity score matched with the RA cohort by age (every 5-year span), gender, index year, urbanization, occupation, and comorbidities. OA was defined by ICD-9-CM code: 715. xx. In order to improve the accuracy of the diagnosis of OA, only patients who had been hospitalized with OA were included. Patients younger than 20 years of age and diagnosed with OA before the index date were excluded.

Comorbidities were considered as important confounding factors, the comorbidities included hypertension (ICD-9-CM code: 401−405), diabetes (ICD-9-CM code: 250), hyperlipidemia (ICD-9-CM code: 272), COPD (ICD-9-CM code: 491, 492, 496), chronic liver diseases (ICD-9-CM code: 571.0, 571.1, 571.2, 571.3, 571.40, 571.41, 571.49, 571.5, 571.6, 571.8, 571.9, V02.61, 070.20, 070.22, 070.30, 070.32, V02.62, 070.41, 070.44, 070.51, 070.54), and gout (ICD-9-CM code: 274). All study subjects were observed until OA occurred, death, withdrawal from NHIRD, or December 31, 2013.

### Statistical Analysis

In this study, we presented gender, age, urbanization, occupation, and comorbidities in a RA cohort and a non-RA cohort based on number and percentage. The mean age was described by mean and standard deviation. The difference of each variable in RA and the comparison cohort were calculated by standardized mean differences (SMD). A value <0.1 indicated that the difference between the two groups was negligible.

To estimate the risk of OA in a RA cohort and the comparison cohort, we used the Cox proportional hazard model and showed the results by hazard ratio (HR) and adjusted hazard ratio (aHR). The cumulative incidence curves of the two groups were plotted by the Kaplan Meier method and statistical significance was checked by the Log-rank test. A two-tailed *p* < 0.05 was considered statistically significant. All statistical analyses in this study were analyzed using SAS statistical software version 9.4 (SAS Institute Inc., Cary, NC). The figure of cumulative incidence curve was plotted by R software.

### Sensitivity Analysis

In the sensitivity analysis, we applied datasets from the LHID, which included one million randomly selected patients in the database of NHI. The difference between this dataset and the inpatients file is that the dataset includes information about drug use and a record of outpatient visits. In order to compare the two, we attempted to make the design of the studies identical. As a starting point, the definition of RA was based on the diagnosis of RA (ICD-9-CM code: 714.0) with the additional criteria of a catastrophic illness card. As an end point, the definition of osteoarthritis was based on the diagnosis of osteoarthritis (ICD-9-CM code: 715. Xx), with at least two outpatient visits or one inpatient visit.

We modified gender, age, urbanization, occupation, comorbidities, and medications for RA in the Cox proportional hazard model, for these factors might be significant confounding factors. Modified comorbidities were the same as the previous model, and the definition of having had comorbidities was based on the following criteria: (1) A record of being diagnosed with a comorbidity before the index date (2) With at least two outpatient visits or one inpatient visit. We also modified drugs for RA in this model, including DMARDs (ATC codes, L01BA01, A07EC01, L04AD01, P01BA02, M01CC01, L04AA13), NSAIDs (ATC code, M01A), steroids for RA (ATC codes, H02AB04, H02AB06), and Biologic therapies (ATC codes, L04AB04, L04AB01, L04AB02, L01XC02, L04AA24, L04AC07). The definition of taking RA drugs was determined based on the record of received prescription drugs in the period of the present study.

## Results

### Enrollment and Patient Characteristics

To explore the association between RA and OA, we recruited a total of 63,626 subjects; of which 31,813 were RA patients and the rest were non-RA patients (Table 1). In this study, about 77% were women and 23% were men. The mean ages of the non-RA and RA cohorts were 53.48 and 53.31 years, respectively. After propensity score matching, there was no significant difference between the two cohorts in each of the variables mentioned above.

**Table 1:** Demographic characteristics and comorbidities of patients newly diagnosed Rheumatoid arthritis (RA) in Taiwan during 2000–2012.

**Characteristics**	**Total**	**Rheumatoid arthritis**		**Standardized mean differences^**§**^**
		**No** ***n* = 31,813**	**Yes** ***n* = 31,813**	
	**N**	**n (%)/** **mean ± SD**	**n (%)/** **mean ± SD**	
**Gender**				
Female	48,848	24,313 (76.4)	24,535 (77.1)	0.017
Male	14,778	7,500 (23.6)	7,278 (22.9)	0.017
**Age**				
20–39	10,691	5,296 (16.6)	5,395 (17.0)	0.008
40–64	39,572	19,753 (62.1)	19,819 (62.3)	0.004
≥65	13,363	6,764 (21.3)	6,599 (20.7)	0.013
Mean (SD)^§^		53.48 ± 13.93	53.31 ± 13.74	0.012
**Urbanization**				
I (highest)	19,654	9,874 (31.0)	9,780 (30.7)	0.006
II	18,935	9,459 (29.7)	9,476 (29.8)	0.001
III	10,494	5,217 (16.4)	5,277 (16.6)	0.005
IV	14,543	7,263 (22.8)	7,280 (22.9)	0.001
**Occupation**				
Office workers	32,173	15,997 (50.3)	16,176 (50.8)	0.011
Manual workers	26,457	13,286 (41.8)	13,171 (41.4)	0.007
Others	4,996	2,530 (8.0)	2,466 (7.8)	0.007
**Baseline comorbidity**				
Hypertension	6,131	3,285 (10.3)	2,846 (8.9)	0.047
Diabetes mellitus	3,732	2,075 (6.5)	1,657 (5.2)	0.056
Hyperlipidemia	1,738	965 (3.0)	773 (2.4)	0.037
COPD	1,304	694 (2.2)	610 (1.9)	0.019
Chronic liver disease	3,215	1,803 (5.7)	1,412 (4.4)	0.056
Gout	1,736	909 (2.9)	827 (2.6)	0.016
Hospitalization, times		4.0 ± 5.0	4.8 ± 5.6	0.151

§ *A standardized mean difference of ≤ 0.1 indicates a negligible difference between the two cohorts*.

### Risk Analysis for Developing of OA in RA Patients

Table 2 shows the adjusted hazard ratio of OA occurring in the RA and non-RA cohorts determined by the Cox proportional hazard model after controlling all other variables. Our results show that patients with RA had greater risk of developing of OA. In patients without RA as a reference group, the risk of OA in RA patients was 2.75 times higher than in patients without RA (95% confidence interval: 2.52–2.99). Examining other variables, the risk of developing OA in men is 0.71 times than that of women (95% CI: 0.64–0.78). The risk of OA among patients aged 40–64 was higher than those patients aged 20–39 (aHR = 3.61). For patients over 65 years of age, the risk of OA was 7.00 times higher than that of patients aged 20–39 (95% CI: 5.80–8.44). The lowest level of urbanization was associated with a high risk of OA (aHR = 1.25). The OA risk of manual workers was 1.32 times higher than that of office workers (95% CI: 1.21–1.43). The presence of baseline comorbidities was associated with an increased risk of OA. The diagnosis of hypertension (aHR = 1.21, 95% CI: 1.07–1.36) and gout (aHR = 1.39, 95% CI: 1.16–1.67) increased the risk of OA in the RA cohort. However, those diagnosed with diabetes mellitus (aHR = 0.86, 95% CI: 0.74–1.01), hyperlipidemia (aHR = 1.10, 95% CI: 0.90–1.34), COPD (aHR = 0.95, 95% CI: 0.76–1.18), and chronic liver disease (aHR = 0.85, 95% CI: 0.72–1.01) with the diagnosis of RA had no significantly higher risk compared to the non-RA cohort.

**Table 2:** Cox model measured hazard ratio and 95% confidence intervals of osteoarthritis associated with and without Rheumatoid arthritis (RA) patients.

**Characteristics**	**Event**	**Person**	**IR**	**Crude**		**Adjusted**	
	**(*n* = 2,736)**	**year**		**HR (95% CI)**	***p*-value**	**HR (95% CI)**	***p*-value**
**Rheumatoid arthritis**							
No	726	217,849	3.33	Ref.		Ref.	
Yes	2,010	211,011	9.53	2.86 (2.63–3.11)	<0.001	2.75 (2.52–2.99)	<0.001
**Gender**							
Female	2,214	338,542	6.54	Ref.		Ref.	
Male	522	9,0318.2	5.78	0.88 (0.80–0.97)	0.010	0.71 (0.64–0.78)	<0.001
**Age at baseline**							
20–39	128	78,688.1	1.63	Ref.		Ref.	
40–64	1,626	274,336	5.93	3.65 (3.05–4.37)	<0.001	3.61 (3.01–4.32)	<0.001
≥65	982	75,835.8	12.95	8.01 (6.66–9.63)	<0.001	7.00 (5.80–8.44)	<0.001
**Urbanization**							
I (highest)	712	131,497	5.41	Ref.		Ref.	
II	763	128,329	5.95	1.10 (0.99–1.22)	0.070	1.02 (0.92–1.13)	0.736
III	435	70,724.8	6.15	1.14 (1.01–1.28)	0.036	1.14 (1.02–1.29)	0.027
IV	826	98,309.3	8.40	1.55 (1.40–1.72)	<0.001	1.25 (1.13–1.39)	<0.001
**Occupation**							
Office workers	1,103	219,818	5.02	Ref.		Ref.	
Manual workers	1,427	176,434	8.09	1.61 (1.49–1.74)	<0.001	1.32 (1.21–1.43)	<0.001
Others	206	32,608.2	6.32	1.26 (1.09–1.46)	0.002	1.10 (0.94–1.27)	0.235
**Baseline comorbidity**							
Hypertension	408	31,242	13.06	2.23 (2.01–2.48)	<0.001	1.21 (1.07–1.36)	0.002
Diabetes mellitus	208	18,952.8	10.97	1.78 (1.54–2.05)	<0.001	0.86 (0.74–1.01)	0.058
Hyperlipidemia	118	8,818.08	13.38	2.14 (1.78–2.58)	<0.001	1.10 (0.90–1.34)	0.370
COPD	91	6,265.84	14.52	2.31 (1.87–2.85)	<0.001	0.95 (0.76–1.18)	0.653
Chronic liver disease	162	17,201.4	9.42	1.50 (1.28–1.76)	<0.001	0.85 (0.72–1.01)	0.063
Gout	138	9,020.34	15.30	2.46 (2.08–2.92)	<0.001	1.39 (1.16–1.67)	<0.001

*HR, hazard ratio; CI, confidence interval; IR, incidence rates, per 1,000 person-years*.

*Adjusted HR, adjusted for gender, age, urbanization, occupation, hospitalization times and all comorbidities in Cox proportional hazards regression*.

Table 3 shows the sites of osteoarthritis in all the involved patients. Over half of the tracked patients had their osteoarthritis on their lower leg, with the ratio of 65.9 percent. Pelvic region and thigh was also a popular site for the occurrence of osteoarthritis, with 11.4% of patients suffering OA in that region.

**Table 3:** Sites of Osteoarthritis in the model of impatient file.

**Sites**	***n***	**%**
Generalized	11	0.4
Shoulder region	32	1.2
Upper arm	16	0.6
Forearm	20	0.7
Hand	27	1.0
Pelvic region and thigh	311	11.4
Lower leg	1,802	65.9
Ankle and foot	52	1.9
Unspecified	465	17.0
Total	2,736	100.0

**Figure 1:**
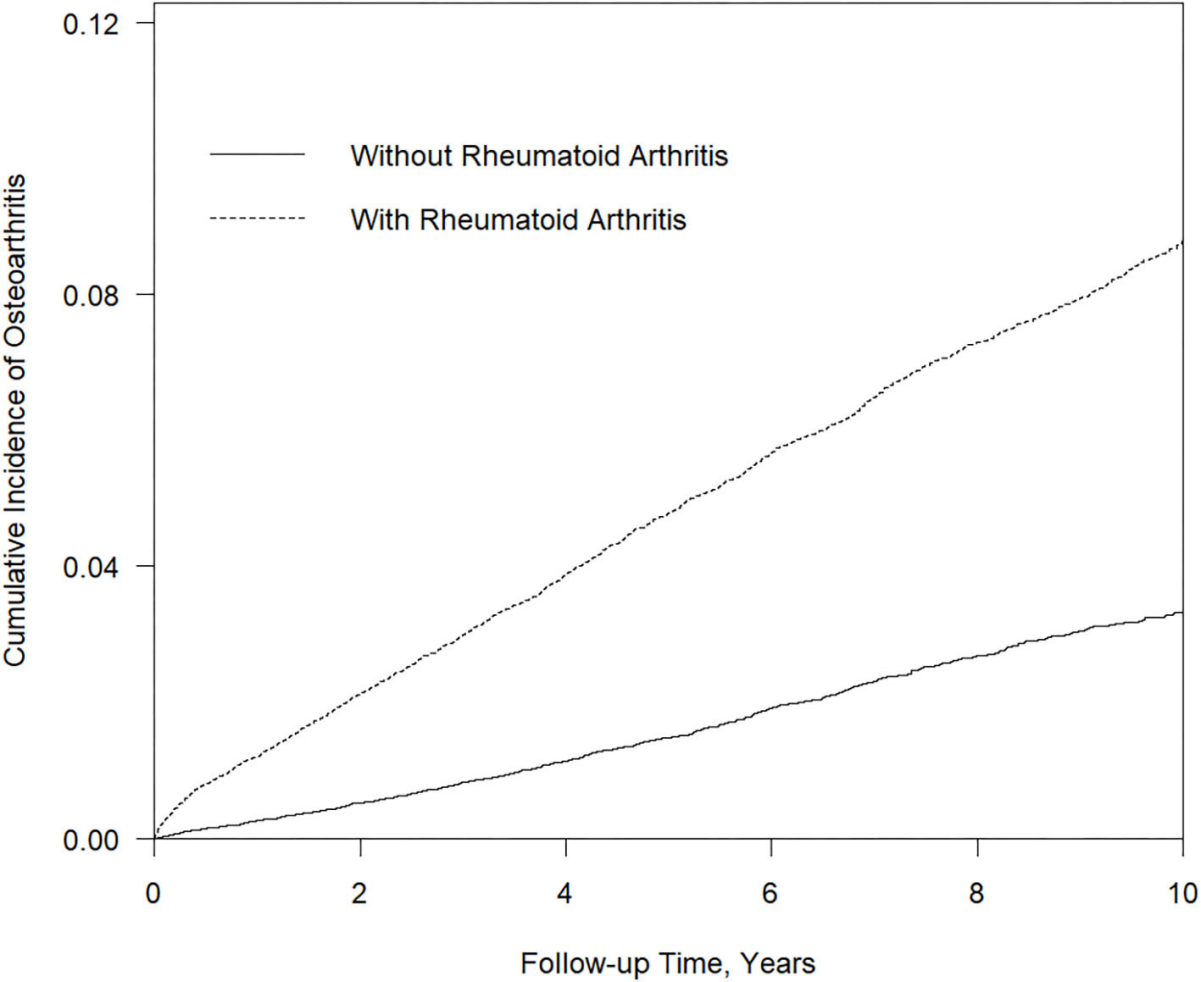
Kaplan Meier survival curves followed up for 10 years in RA and non-RA cohorts.

Tables 4–6 demonstrated sensitivity analysis results. The baseline characteristic of patients in this model is shown in Table 4. The ratio of female and male patients was ~77 and 23%, respectively. The mean ages of the non-RA cohorts were 47.9 years and the RA cohorts 46.6 years. Through propensity score matching, the difference between variables in the two cohorts of this model has been eliminated. Table 5 showed the Cox proportional hazard model. In the LHID dataset, the incidence rate of RA patients developing subsequent osteoarthritis was 45.70 per 1,000 person-year, and the aHR was 1.43 (95% C.I., 1.06–1.94). Table 6 showed the sites of osteoarthritis in the LHID dataset. About 50 percent of the sites were recorded unspecified. Within all specified sites, the lower leg was the most frequently diagnosed area for osteoarthritis, with a ratio of 30.8 percent. Figure 1 shows that the cumulative incidence of OA in the RA cohort was significantly higher than in the non-RA cohort (*p* < 0.001).

**Table 4:** Demographic characteristics and comorbidities of patients newly diagnosed Rheumatoid arthritis (RA) in Taiwan during 2000–2012, in LHID dataset.

**Characteristics**	**Total**	**Rheumatoid arthritis**		***p*-value**
		**No** ***n* = 412**	**Yes** ***n* = 412**	
	**N**	**N (%)/** **mean ± SD**	**N (%)/** **mean ± SD**	
Gender				0.620
Female	634	314 (76.2)	320 (77.7)	
Male	190	98 (23.8)	92 (22.3)	
Age				0.235
20–39	239	114 (27.7)	125 (30.3)	
40–64	514	256 (62.1)	258 (62.6)	
≥65	71	42 (10.2)	29 (7)	
Mean (SD)^a^		47.9 (13.2)	46.6 (12.5)	0.160
Urbanization				0.767
I (highest)	251	121 (29.4)	130 (31.6)	
II	222	109 (26.5)	113 (27.4)	
III	167	89 (21.6)	78 (18.9)	
IV	184	93 (22.6)	91 (22.1)	
Occupation				0.933
Office workers	492	247 (60)	245 (59.5)	
Manual workers	278	137 (33.3)	141 (34.2)	
Others	54	28 (6.8)	26 (6.3)	
**Baseline comorbidity**				
Hypertension	188	94 (22.8)	94 (22.8)	1.000
Diabetes mellitus	109	57 (13.8)	52 (12.6)	0.607
Hyperlipidemia	185	95 (23.1)	90 (21.8)	0.676
COPD	119	59 (14.3)	60 (14.6)	0.921
Chronic liver disease	180	91 (22.1)	89 (21.6)	0.866
Gout	187	96 (23.3)	91 (22.1)	0.678
**Medication for RA**				
NSAIDs	587	286 (69.4)	301 (73.1)	0.248
Biologic therapies	51	1 (0.2)	50 (12.1)	<0.001
DMARDs	305	5 (1.2)	300 (72.8)	<0.001
Steroids	399	127 (30.8)	272 (66.0)	<0.001

*Chi-square test, t-test^a^*.

**Table 5:** Cox model measured hazard ratio and 95% confidence intervals of osteoarthritis associated with and without Rheumatoid arthritis (RA) patients, in LHID dataset.

**Characteristics**	**Event**	**Person**	**IR**	**Crude**		**Adjusted**	
	**(*n* = 201)**	**year**		**HR (95% CI)**	***p*-value**	**HR (95% CI)**	***p*-value**
**Rheumatoid arthritis**							
No	88	2,479	35.50	Ref.		Ref.	
Yes	113	2,473	45.70	1.29 (0.98–1.71)	0.073	1.43 (1.06–1.94)	0.020
**Gender**							
Female	161	3,906	41.22	Ref.		Ref.	
Male	40	1,046	38.25	0.93(0.66–1.31)	0.674	0.89 (0.62–1.30)	0.556
**Age at baseline**							
20–39	30	1,695	17.70	Ref.		Ref.	
40–64	141	2,967	47.52	2.71 (1.83–4.02)	<0.001	1.56 (1.01–2.42)	0.045
≥65	30	289	103.69	5.99 (3.60–9.99)	<0.001	2.78 (1.58–4.90)	<0.001
**Urbanization**							
I (highest)	56	1,531	36.57	Ref.		Ref.	
II	62	1,312	47.25	1.29 (0.90–1.86)	0.162	1.36 (0.92–2.01)	0.126
III	27	1,012	26.67	0.72 (0.46–1.14)	0.164	0.63 (0.39–1.03)	0.066
IV	56	1,095	51.13	1.39 (0.96–2.01)	0.082	0.67 (0.44–1.03)	0.065
**Occupation**							
Office workers	102	3,024	33.73	Ref.		Ref.	
Manual workers	85	1,597	53.24	1.59 (1.19–2.12)	0.002	0.99 (0.71–1.38)	0.942
Others	14	331	42.31	1.28 (0.73–2.23)	0.393	0.92 (0.51–1.67)	0.790
**Baseline comorbidity**							
Hypertension	64	1,004	63.74	1.83 (1.36–2.46)	<0.001	1.03 (0.72–1.48)	0.870
Diabetes mellitus	34	585	58.16	1.50 (1.04–2.17)	0.031	1.53 (1.02–2.30)	0.042
Hyperlipidemia	64	1,026	62.38	1.78 (1.33–2.40)	<0.001	0.92 (0.64–1.32)	0.642
COPD	29	623	46.53	1.18 (0.80–1.75)	0.408	0.93 (0.60–1.44)	0.740
Chronic liver disease	55	917	59.98	1.67 (1.22–2.28)	0.001	1.55 (1.10–2.18)	0.011
Gout	51	995	51.24	1.33 (0.97–1.83)	0.076	1.46 (1.02–2.10)	0.041
**Medication for RA**							
NSAIDs	6	3,991	1.50	0.01 (0.00-0.02)	<0.001	0.01 (0.00–0.02)	<0.001
Biologic therapies	29	2,800	10.36	0.13 (0.09–0.19)	<0.001	0.62 (0.40–0.96)	0.032
DMARDs	14	2,031	6.89	0.11 (0.06–0.19)	<0.001	0.67 (0.37–1.22)	0.192
Steroids	1	402	2.49	0.06 (0.01–0.42)	0.005	0.65 (0.08–5.01)	0.682

*HR, hazard ratio; CI, confidence interval; IR, incidence rates, per 1,000 person-years*.

*Adjusted for gender, age, urbanization, occupation, comorbidities and medications for RA in Cox proportional hazards regression*.

**Table 6:** Sites of Osteoarthritis in LHID dataset.

**Sites**	***n***	**%**
Generalized	20	10.0
Shoulder region	7	3.5
Upper arm	1	0.5
Forearm	2	1.0
Hand	2	1.0
Pelvic region and thigh	4	2.0
Lower leg	62	30.8
Ankle and foot	3	1.5
Unspecified	100	49.8
Total	201	100.0

## Discussion

There were no previous epidemiological studies on the association between RA and OA. Our longitudinal retrospective cohort study revealed that patients with RA were at higher risk of developing OA after adjusting the demographic factors and comorbidities. The risk of OA in RA patients was 2.75 times higher than in patients without RA. Our study demonstrated that RA was also one of the important risk factors for OA. Given the high disability burden of OA, OA prevention was very important in RA patients.

Current evidence on OA-related risk factors include systemic and local risk factors. Systemic risk factors include social demographics, genetics, obesity, metabolic syndrome, diet, and bone mineral density (15). Older age was a well-known risk factor for OA (16–18). This study found that patients of 40–64 years of age had a higher risk of OA than patients aged 20–39 years (aHR = 3.61). For patients over 65 years of age, the risk of OA is 7.00 times higher than that of patients aged 20–39 years (95% CI: 5.80–8.44). Compared with women, men have less hand, foot and knee OA, but are more likely to develop cervical spine OA (16). Our study found that the risk of developing OA in men is 0.71 times than that of women (95% CI: 0.64–0.78). Several cohort studies also discussed whether occupational activities have an impact on OA. Two studies investigated the heavy physical workload, but both considered it not important (19, 20). However, our study showed that the OA risk of manual workers was 1.32 times higher than that of office workers (95% CI: 1.21–1.43).

Since the population-based inpatient file did not include information about outpatient visits and medications, we conducted the sensitivity analysis utilizing LHID to strengthen our findings. In this LHID model, definition of osteoarthritis does not merely include hospitalization, but also includes outpatient visits. Considering that some patients might be treated in an OPD setting but might never be hospitalized for OA related conditions, the definition in the sensitivity analysis could avoid these patients being excluded from the study. Additionally, in the LHID analysis, RA medication was added as a controlled variable in the Cox proportional hazard model. Although inpatient files were not able to provide information about medication and might cause confounding bias, the model in LHID was able to solve this problem.

In different RA patients, baseline status and sites of osteoarthritis differs. Previously, based on radiographs of patients' hands and feet, a research disclosed that in patients with early RA, osteoarthritis was common at the baseline. (21) However, in the present study, all patients with osteoarthritis were newly-diagnosed, and people diagnosed with osteoarthritis before the diagnosis of RA were excluded from our study. Accordingly, the baseline rate of osteoarthritis in both groups were zero. Examination of the Osteoarthritis Initiative (OAI) data revealed a link between elevated systolic blood pressure and increased incidence of radiographic knee OA (22). Our study showed hypertension increased the risk of OA 1.21 times in the RA cohort. Previous studies had rarely studied the relationship between hyperlipidemia and OA (17). A recent case-control study from the United Kingdom had shown that hyperlipidemia is an independent risk factor for new-onset OA (23). Our study showed hyperlipidemia did not increase the risk of developing OA in the RA cohort. A study shows that respiratory disease is one of the risk factors for OA (24). However, our research does not show that COPD increases the risk of OA in RA populations. Gouty arthritis is a common inflammatory arthritis affecting about 5% of the elderly population worldwide (25). The amount of uric acid in a person's joints may increase the likelihood of OA (26). Uric acid crystals deposited in the cartilage can cause cartilage degeneration, and OA (27). Our study showed gouty arthritis increased the risk of OA 1.39 times in the RA cohort. In addition, some recent reports do not support the association between diabetes and hand/knee OA (28–30). Our study also demonstrated that diabetes (aHR = 0.86, 95% CI: 0.74–1.01) along with a diagnosis of RA had no significantly lower risk compared to the non-RA cohort.

Chronic inflammation was considered a key pathogenesis factor of OA in patients with RA (31–33). Patients with RA exhibit chronic systemic inflammation, which can invade the soft tissues of the joints, such as the bursa and joint capsule which will impair the stability of the joint (34). Joint instability would increase cartilage wear and then OA developed (11, 15). Inflammation can also vitiate the remodeling and healing of the cartilage and increase the vulnerability of RA patients in developing OA lesions. The increased expression of inflammatory cytokines in RA patients was also found in OA patients (31–33). Besides, anti-inflammatory cytokines such as IL-4, IL-10, and IL-13, had been found to be involved in the pathogenesis of OA (13, 14). New understanding on the role of inflammation in both RA and OA has given insights into a possible shared pathogenesis pathway. It revealed a close connection between these two disorders. Hence, the exposure of RA patients to chronic systemic inflammation may contribute to subsequent OA increases.

We used a nationwide, population-based claims database which can help minimize recall and selection bias. The strength of this study was in its large sample size. The sufficiently large sample size and robust analysis lends confidence to the final results. However, this study has some limitations that should be considered. Firstly, the diagnoses of RA, OA, and comorbidities were based on the ICD-9-CM code in the database; hence the accuracy should be addressed. However, to ensure an accurate diagnosis, the Bureau of NHI medical records are regularly reviewed by expert medical specialists. Furthermore, patients with RA in Taiwan can apply for catastrophic illness registration cards, which require approval by the Bureau of NHI before being issued. The above measures can ensure the accuracy of the diagnosis. Secondly, since our data sources were obtained from a secondary database, radiographic reports, serological data (including inflammatory markers), and lifestyle factors (for example, smoking, diet, BMI, and physical activity) were not available, and could not be included in the study. Finally, in the model using inpatient files, the influence of RA related medication such as steroids, NSAIDs, DMARDs, and biologic therapies, which will affect the progression of the disease were not analyzed. However, in the sensitivity analysis, the RA related medication has been adjusted to minimize the influence of this possible confounding factor.

## Conclusion

This is a robust large-scale cohort study to investigate the risk of OA among patients with RA. Our study indicates that during the 13-year longitudinal follow-up period, RA patients were at a higher risk of being diagnosed with OA than the control cohort. Developing effective OA prevention strategies are necessary in patients with RA. This study may be extended to evaluate the risk of OA among other kinds of inflammatory autoimmune diseases. Identifying the key pathogenesis mechanisms are necessary for future study.

## Data Availability Statement

The raw data supporting the conclusions of this article will be made available by the authors, without undue reservation.

## Ethics Statement

The studies involving human participants were reviewed and approved by MOHW108-TDU-B-212-133004. Written informed consent for participation was not required for this study in accordance with the national legislation and the institutional requirements.

## Author Contributions

Y-HL and H-KT contributed to study design, grant, drafted the initial manuscript, reviewed, and revised the manuscript. S-LK contributed to administrative works. S-YG and Y-CB contributed to revise the manuscript and reply letter. M-CL contributed to analyze data. JW and H-KT contributed to the study design, reviewed, and revised the manuscript. All authors contributed to the article and approved the submitted version.

## Conflict of Interest

The authors declare that the research was conducted in the absence of any commercial or financial relationships that could be construed as a potential conflict of interest.
